# Evaluation of Rheological Properties of Asphalt Binder Modified with Biochar from Oat Hulls

**DOI:** 10.3390/ma17174312

**Published:** 2024-08-30

**Authors:** Camila Martinez-Toledo, Gonzalo Valdes-Vidal, Alejandra Calabi-Floody, María Eugenia Gonzalez, Oscar Reyes-Ortiz

**Affiliations:** 1 Engineering Doctoral Program, Universidad de La Frontera, Temuco 4811230, Chile; c.martinez23@ufromail.cl; 2 Department of Civil Engineering, Universidad de La Frontera, Temuco 4811230, Chile; alejandra.calabi@ufrontera.cl; 3 Department of Chemical Engineering, Universidad de La Frontera, Temuco 4811230, Chile; mariaeugenia.gonzalez@ufrontera.cl; 4 Department of Civil Engineering, Military University of Nueva Granada, Bogotá 111711, Colombia; oscar.reyes@unimilitar.edu.co

**Keywords:** biochar, asphalt binder, rheological properties

## Abstract

In this study, the effect of biochar from oat hulls (BO) on the rheological properties of a PG 64-22 asphalt binder was evaluated using a full factorial design, which included the following factors: pyrolysis temperature (PT) (300 °C and 500 °C), BO particle size (<20 µm and <75 µm), and the amount of BO (2.5%, 5%, and 7.5%). First, the morphological and physicochemical properties of BO were analyzed by comparing it with graphite powder (CFG) and commercial activated carbon (CAC). The physicochemical properties of the modified asphalt binder were then evaluated using confocal laser microscopy, scanning electron microscopy (SEM–EDX), and Fourier-transform infrared spectroscopy (FTIR). Its storage stability was also evaluated. Subsequently, the rutting parameter G*/sin(δ) and the Fraass breaking point were analyzed to select asphalt binders that extended their viscoelastic range. The asphalt binders selected were those with 2.5%, 5%, and 7.5% BO, produced at a PT of 300 °C with a particle size <20 µm (BO300S). Next, the rheological properties of the selected samples were evaluated by testing for rotational viscosity, rutting parameter G*/sin(δ), multiple stress creep recovery (MSCR), fatigue parameter G*·sin(δ), and creep stiffness by bending beam rheometry (BBR). The rheological aging index according to rutting parameter G*/sin(δ) (RAI) was also evaluated. These tests were conducted in different states of the asphalt binder: original, short-term aged, and long-term aged. According to the results, the application of BO300S significantly increased the resistance of the asphalt binder to rutting and rotational viscosity, proportional to the amount added to the asphalt binder. Moreover, low modifier percentages improved fatigue resistance, outperforming CFG and CAC. In addition, it performs well at low service temperatures, registering better resistance than the control asphalt binders.

## 1. Introduction

According to recent estimates, about 200 million tons of asphalt binder are produced annually, 85% of which is used for the construction of asphalt pavements because it is the most widely used type of road infrastructure worldwide [1,2,3,4,5,6,7].

Currently, economic development and population growth have encouraged the construction of new asphalt pavements and the maintenance of existing ones [8]. This has become a problem due to the decrease in oil reserves and the pollution generated during asphalt binder production due to greenhouse gases (GHGs) [8,9,10,11,12]; therefore, new materials or materials from other industries must be incorporated to make roads more durable and sustainable.

Thus, asphalt binder modifiers are also being used to improve their performance under existing climate and traffic conditions [13]. According to the literature, nano- and micrometer-sized carbonaceous materials have been successfully used as alternative commercial modifiers, including biochar [11,14,15,16]. Biochar is one of the by-products of pyrolysis [17], a thermochemical process that occurs between 300 °C and 1000 °C under oxygen-limited conditions, from which the other by-products, syngas and bio-oil, are also obtained [18,19,20]. Biochar is a solid, carbon-rich material, which, when pyrolyzed at lower temperatures (between 300 °C and 500 °C), achieves a higher production yield [21,22,23]. Among the raw materials used for its generation are wood-based products (such as wood chips, bark, and sawdust), organic and industrial wastes (such as sludge and manure), and plant materials (such as leaves, hulls, seeds, cobs, and others) [24]. Depending on the raw material used and the pyrolysis operating conditions, the resulting biochar can have favorable physicochemical properties to improve the eco-environmental quality in various fields [25]. Accordingly, most studies have focused on its use to enhance the quality of soil for crops due to its benefits: waste reuse, fertility improvement, and carbon sequestration [24,26,27]. On the other hand, biochar has also been used in construction materials (e.g., concrete), with only a few studies on this subject [28,29].

Recent studies have shown favorable results concerning the use of biochar as an asphalt binder modifier. For example, biochar derived from pig manure improved the performance of the asphalt binder at high service temperatures and decreased its oxidation rate when added between 2% and 10% (by weight) to the asphalt binder [30]. In addition, studies have been conducted on the effect of particle size and the production process of biochar from wood waste on the rheological properties of the asphalt binder, indicating that particles <75 µm improved fatigue and cracking resistance [9,31]. On the other hand, studies have also been conducted where biochar and bio-oil from wood waste were mixed to modify the asphalt binder, indicating that this mixture worked as a rejuvenating additive [31,32]. 

According to the study conducted by González et al. [33], it is known that the properties of biochar are largely affected by the type of raw material and pyrolysis operating conditions. In that sense, they determined that BO achieves a better productive yield through slow pyrolysis with a PT of 300 °C and obtains a high carbon content, low heavy metal content, and varied functional groups. On the other hand, Martínez-Toledo et al. [34] evaluated the use of pyrolyzed BO at 300 °C as a modifier of the physical or conventional properties of the CA–24 asphalt binder (according to the Chilean specification [35]), indicating that particles <75 µm were homogeneously distributed in the asphalt binder and that it improved the rotational viscosity, penetration, and softening point. This could be attributed to chemical interactions between the asphalt binder and BO via ―C=C and ―C=O bonds. In addition, since the asphalt binder also has a carbonaceous elemental chemical composition (between 82% and 88%), the interactions of the polar or polarizable composition of both materials could have favorable effects on the rheological properties of the asphalt binder [1,36,37]. Based on this information, it is clear that other influential variables of BO on the asphalt binder have not been studied, such as particle sizes below 75 µm and the effect of other PTs on its morphological and physicochemical properties. Its impact on the rheological properties of asphalt binders has also not been studied. 

In the context of oats, according to the records of the United States Department of Agriculture (USDA) and the Food and Agriculture Organization Corporate Statistical Database (FAOSTAT), during the last six seasons, the surface area of oat crops exceeded 9.8 million hectares and a production of nearly 23 million tons worldwide, with Russia, the European Union, Canada, the United States, and Australia accounting for 78.6% of the world’s crops [38,39]. Traditionally, oats have been used as a food product [40,41]. For this reason, groat (peeled and stabilized grain) is the main export product (~43%), followed by flakes (~38%) and raw oats (oats with the hull used for animal consumption) (~15%) [38,42]. This means large volumes of residual biomass are generated from the hull alone [43]. This constitutes approximately 30% of the total weight of the cereal [44], although some authors argue that this value could range between 25% and 36%, making it a constant problem for millers [43,45,46,47]. The first uses of oat hulls were as a source for the production of furfural, a renewable material used for adhesives, plastics, and nylon [43,48,49]. However, it was replaced by corn cobs and sugarcane bagasse [43]. Currently, the most common uses are combustion biomass for electricity and steam generation [50] and as a source of fiber for supplements and animal feed [43,49]. 

In that sense, BO could be used as a potential asphalt binder modifier. Valorizing its hull in the road paving industry could contribute to advancing the circular economy of the oat agricultural industry. In addition, it would provide a more environmentally friendly solution for the need to improve the strength and durability of asphalt pavements.

For these reasons, this study aimed to analyze the effect of BO on the rheological properties of the asphalt binder at different service temperatures. This would establish its resistance to the usual deteriorations in asphalt pavements, such as rutting, aging, fatigue, and thermal cracking.

## 2. Materials and Methods

### 2.1. Materials

The asphalt binder used in this study is a PG 64–22 type, classified according to the Superpave methodology. Its properties are shown in Table 1.

**Figure 1:**
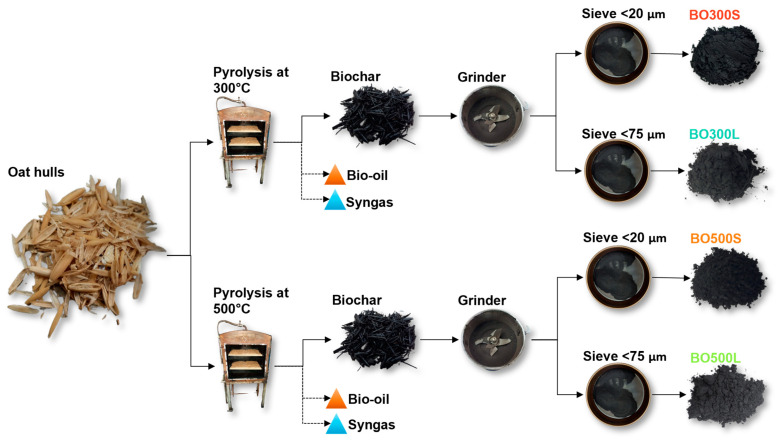
BO production. “BO300S”: BO pyrolyzed at a PT of 300 °C with a particle size <20 µm. “BO300L”: BO pyrolyzed at a PT of 300 °C with a particle size <75 µm. “BO500S”: BO pyrolyzed at a PT of 500 °C with a particle size <20 µm. “BO500L”: BO pyrolyzed at a PT of 500 °C with a particle size <75 µm.

BO was obtained from the *sativa L*. species, where 80% corresponded to the supernova variety and 20% to the Uranus variety, whose chemical composition is characterized by a high percentage of carbon and hydrocarbon functional groups [33,56]. Its production was through slow pyrolysis in an electric pyrolysis furnace with a capacity of 3 kg per batch. The pyrolysis was programmed using a programmable logic controller (PLC), considering 2 h of residence time with a heating rate of 3.6 °C/min and a PT of 300 °C and 500 °C, respectively. In addition, nitrogen (N_2_) was used at a flow rate of 0.001 m^3^/min to purge the oxygen generated during the reactions. BO production was similar for both PTs, with an approximate yield equivalent to 45% by weight. When BO was cooled, it was ground using a blade grinder at 28,000 r/min for 30 s and then sieved to obtain particle sizes <20 µm and <75 µm for each PT (Figure 1). CFG and CAC were used as commercial modifier controls for comparison purposes. These have improved the rheological properties of asphalt binders at different service temperatures and aging conditions according to modification methods and tests similar to those proposed in this study [57,58,59,60,61,62,63,64,65]. CFG and CAC were used with particle sizes of <75 µm.

### 2.2. Full Factorial Design

The 2 × 2 × 3 full factorial design shown in Table 2 was used to study the combinations formed by the different factor levels simultaneously. This design makes it possible to understand how the factors interact. In addition, it can describe the overall behavior of the experiment and find the optimal combination of levels for the response variable [66]. This factorial design evaluated the rutting parameters G*/sin(δ) and the Fraass breaking point indicating the transition from the viscoelastic to elastic state of the asphalt binder. From these results, asphalt binder samples with BO that tended to increase their viscoelastic range were chosen, and other rheological properties were evaluated at different service temperatures.

The 2 × 2 × 3 full factorial design (Table 2) consists of 12 runs and includes the following factors and levels: PT, 300 °C and 500 °C; particle size, <20 µm and <75 µm; and the amount of BO, 2.5%, 5%, and 7.5%, by the weight of the asphalt binder. These factors and their respective levels were chosen according to previous research where the effect of BO on the physical properties of the asphalt binder was evaluated [34]. In addition, information from other studies on the use of biochar from other raw materials was considered to modify the asphalt binder [8,9,10,22,30,67,68,69].

The following control samples were included for comparison purposes: “AB” asphalt binder; “AB-0.0” asphalt binder with 0% BO but subjected to the modification procedure; “AB-CFG5.0” asphalt binder with 5% CFG; and “AB-CAC5.0” asphalt binder with 5% CAC. All modifiers were added in the percentage indicated for the weight of the asphalt binder.

### 2.3. The Modification of the Asphalt Binder

Prior to modification, the asphalt binder was conditioned in an oven at 140 ± 5 °C for 2 h. Meanwhile, BO was subjected to 120 ± 5 °C for 2 h to remove the moisture absorbed during storage [9,11]. After conditioning, the asphalt binder was modified according to the full factorial design shown in Table 2, using an electronic blade agitator at 160 ± 5 °C for 30 min of mixing at a constant speed of 350 r/min. The samples were then reconditioned in an oven at 120 ± 5 °C for an additional 6 h to promote the adsorption of the asphalt binder by the BO particles [69]. Finally, the modified asphalt binder was stored in metal containers at room temperature. The same modification procedure was used for the control samples: AB-0.0, AB-CFG5.0, and AB-CAC5.0.

### 2.4. Test Methods

#### 2.4.1. Morphological Characterization of BO and Commercial Modifier Controls (CFG and CAC)

The morphological characterization of BO, CFG, and CAC was performed to identify their main differences and similarities. First, the morphology of the particles was determined by scanning electron microscopy (SEM), obtaining high-resolution images of the surface of the samples. Additionally, quantifiable parameters typical of porous materials were obtained, such as BET (Brunauer, Emmett, Teller) surface area (S_BET_), pore volume (V_P_), and pore diameter (D_P_) using a porosimeter. Finally, particle sizes (<20 µm and <75 µm) and their descriptive parameters were determined using the laser diffraction technique, recording the size as the particle diameter.

#### 2.4.2. Physicochemical Characterization of Modifiers and Modified Asphalt Binder

Different physicochemical characterization techniques were used to identify possible interaction mechanisms between the modifiers and the asphalt binder. The modifiers and the asphalt binder were analyzed separately and as modified asphalt binders. Among the analyses performed was the determination of the chemical composition of the modifiers and the BO-modified asphalt binder using a scanning electron microscope equipped with an energy-dispersive X-ray detector, SEM–EDX (SU3500, Hitachi High-Technologies Corporation, Tokyo, Japan). Additionally, the functional groups of the samples were identified by Fourier-transform infrared spectroscopy, FTIR (Cary 630 FTIR, Agilent Technologies, Santa Clara, CA, USA), obtaining the FTIR spectra between the bands located at 4000 cm^−1^ and 600 cm^−1^. The distribution of BO particles in the asphalt binder was also determined by confocal laser microscopy. 

Finally, the storage stability of the BO-modified asphalt binders was evaluated as an additional characterization parameter according to ASTM D5892 [70]. This procedure consisted of conditioning the samples at 163 ± 5 °C for 48 h and then at −6.7 ± 5 °C for 4 h. Subsequently, the softening point test was performed on the top and bottom of each asphalt binder evaluated according to ASTM D36–76 [71].

#### 2.4.3. The Evaluation of the Rheological Properties of the Modified Asphalt Binder

The asphalt binder samples with BO defined by the 2 × 2 × 3 full factorial design in Table 2 were evaluated at both high and low temperatures to establish their range of viscoelastic behavior. The rutting parameter G*/sin(δ) described in the AASHTO T 315 [53] standard was used to evaluate the behavior at high service temperatures. This parameter was determined using a dynamic shear rheometer (DSR) on short-term aged specimens using a rolling thin-film oven (RTFO), and the response variable was the rutting parameter G*/sin(δ) measured in kPa. In contrast, the evaluation of the behavior at low service temperatures was assessed using the Fraass breaking point test, described in UNE-EN 12593:2007 [72]. The test was performed using the Control Breaking Point equipment on long-term aged samples in the pressure aging vessel (PAV) since this is the most unfavorable performance condition for behavior at low temperatures. The bending cycles were performed at decreasing temperatures with a cooling rate of 1 °C/min, and the response variable was the Fraass breaking point measured in °C.

Short-term aging was performed per AASHTO T 240 [54] by subjecting the asphalt binder samples to 163 °C for 85 min in the RTFO. Conversely, long-term aging was performed according to AASHTO R 28 [73], where asphalt binders aged in the RTFO were subsequently subjected to a pressure of 2.1 MPa for 20 h in the PAV. 

From the results obtained from the factorial design, asphalt binder samples with BO that tended to increase their viscoelastic range were selected, and other rheological properties were evaluated at different service temperatures. These are presented next:

The flow resistance and workability were evaluated through rotational viscosity using the Brookfield rotational viscometer according to the procedure described in AASHTO T 316 [52]. This rheological parameter was determined in the original state of the asphalt binder at the following temperatures: 120 °C, 135 °C, 150 °C, and 165 °C.

The rutting resistance was evaluated using rheological parameters related to the high service temperatures of the pavement and determined by a DSR according to the procedures described in AASHTO T 315 [53] and AASHTO T 350 [74], respectively. AASHTO T 315 [53] makes it possible to determine the complex modulus (G*) and phase shift angle (δ) parameters of the asphalt binder in the original and short-term aged state (RTFO). This procedure was performed at 58 °C, 64 °C, 70 °C, and 76 °C to determine the rutting parameter G*/sin(δ). In addition, an angular frequency of 10 rad/s was used, which simulates the shear action corresponding to a vehicle speed of 90 km/h [1]. On the other hand, the AASHTO T 350 [74] standard describes the multiple stress creep recovery (MSCR) test. This test determines additional parameters to those of the Superpave classification related to the load level to which the pavement will be subjected; it is highly recommended for observing the effect of modifiers on the asphalt binder [75]. The MSCR test was used to determine the cumulative deformation, the recovery percentage (R%), and non-recoverable creep compliance (Jnr) under different stress levels (0.1 kPa and 3.2 kPa). This test was performed by a DSR on asphalt binder samples aged short-term in the RTFO at 70 °C.

The fatigue strength was evaluated by the rheological parameter G*·sin(δ) using a DSR. The test was performed by the PAV according to the procedure described in AASHTO T 315 [53] at 16 °C, 19 °C, 22 °C, 25 °C, 28 °C, and 31 °C on the long-term aged samples.

Finally, the susceptibility to thermal cracking was evaluated using the procedure described in AASHTO T 313 [55] on creep stiffness using bending beam rheometry (BBR). In this case, creep stiffness (S) and m-value parameters were analyzed at −6 °C and −12 °C. This indicated the effect of the modifiers on the ability of the asphalt binder to decrease its stiffness when subjected to a 100 g load at low temperatures.

#### 2.4.4. Evaluation of Susceptibility to Aging of Modified Asphalt Binder

The susceptibility to aging was evaluated through the rheological aging index of the rutting parameter G*/sin(δ) (RAI). Low RAI values indicate low susceptibility to aging and, therefore, an asphalt binder with improved aging resistance [76]. The RAI was evaluated between temperatures of 58 °C and 76 °C, using Equation (1):

(1) $$RAI=\frac{{{(G}^{*}/sin(\delta ))}_{RTFO}}{{\left(G^{*}/sin(\delta )\right)}_{original}}$$

where RAI is the rheological aging index of the rutting parameter of (G*/sin(δ))_RTFO_ and (G*/sin(δ))_original_, after and before short-term aging in the RTFO, respectively. 

## 3. Results

### 3.1. Morphological Characterization of BO and Commercial Modifier Controls

**Figure 2:**
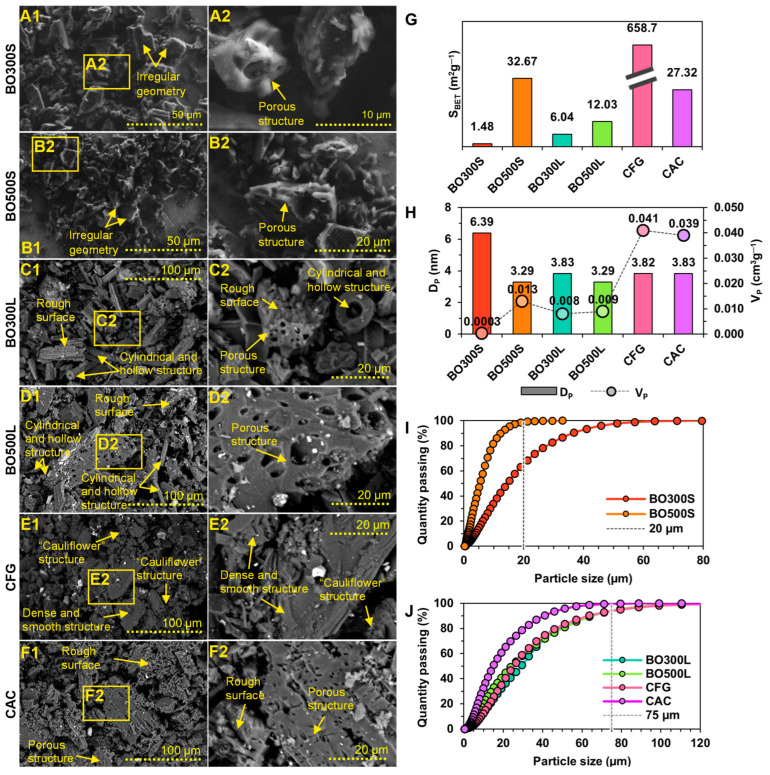
Morphological characterization. (**A1**,**A2**) are SEM micrographs of BO300S. (**B1**,**B2**) are SEM micrographs of BO500S. (**C1**,**C2**) are SEM micrographs of BO300L. (**D1**,**D2**) are SEM micrographs of BO500L. (**E1**,**E2**) are SEM micrographs of the CFG. (**F1**,**F2**) are SEM micrographs of the CAC. Yellow-edged rectangles indicate micrographs at higher magnification, and yellow arrows indicate particle characteristics. (**G**) is the S_BET_ surface area graph. (**H**) is the graph of D_P_ versus V_P_. (**I**) is the graph of particle size distribution <20 µm. (**J**) is the graph of particle size distribution <75 µm.

The importance of the analysis of the morphological properties of the modifiers lies in the fact that they could influence the interaction and homogenization with the asphalt binder, which could cause a variation in its shear strength and performance after the modification procedure [69]. In that sense, the SEM A1–F2 micrographs in Figure 2 show the microscopic morphology of the modifiers used in this study. These figures illustrate that the characteristics of BO300S and BO500S are similar, showing irregular geometries due to the grinding process to obtain particle sizes <20 µm. At higher magnification, BO300S and BO500S have particles with porous structures, the pores of which could contribute to a larger BO surface area. According to the literature, the development of pores on the BO surface is due to the pyrolysis conditions, such as residence time and PT, as well as the raw material used, i.e., oat hulls [33,56]. In the case of BO300L and BO500L, particles with porous structures composed of different pore diameters are noted. This condition could improve the interaction with the asphalt binder, contributing to enhancing the resistance of the asphalt binder and asphalt mixture to plastic deformations generated at high service temperatures in a pavement [9,11,22,67,68]. Other particles are also found in BO300L and BO500L, with more varied microscopic morphologies than in the smaller BO (<20 µm) and the commercial modifier controls. For example, particles with cylindrical and hollow structures are observed that, despite being larger than 75 µm, pass through the sieve mesh due to their narrow and elongated shape. Particles with rough surfaces are also observed, which become more complex towards a PT of 500 °C. Compared to commercial modifier controls, CFG has structures different from those of BO, such as particles with dense structures and smooth surfaces. In addition, it has particles with “cauliflower” structures formed by carbon layers with curved and discontinuous shapes, typical of synthetic graphite [22,60,77,78]. In contrast, CAC is similar to BO, with particles with porous structures and rough surfaces. However, CAC pores apparently have similar diameters and are more evenly distributed over the particle surface [9,63].

Figure 2G,H provide the results obtained for the S_BET_, D_P_, and V_P_ of the BO and the commercial modifier controls. The data shown in Figure 2G suggest that the S_BET_ of BO is higher when pyrolysis is performed at a higher PT (500 °C). This was observed for both particle sizes; for example, in those <20 µm, BO500S had a higher S_BET_ than that of BO300S at ~31 m^2^g^−1^. Meanwhile, in particles <75 µm, BO500L had a higher S_BET_ than BO300L at ~6 m^2^g^−1^, corresponding to practically twice the value. This indicates a correlation between the specific surface area of BO and the temperature at which pyrolysis occurs. In this sense, higher PTs likely influence the plastic deformations generated in the raw material during biochar production, promoting the development of particles with a porous structure [79,80,81]. CFG presents an S_BET_ considerably higher than that of BO. This may be due to their “cauliflower” structures, which generate very rough particles and are also included in the S_BET_ calculation. On the other hand, a comparison of the BO and CAC results shows that the S_BET_ of most of the BO is lower than that of CAC. This is unlike BO500S, which has an S_BET_ 19% higher than that of CAC, attributed to the heterogeneity of the diameter and pore volume of BO500S particles.

According to the D_P_ and V_P_ results (Figure 2H), it cannot be ruled out that all the modifiers used in this study have particles with porous structures, although, in some SEM micrographs, they cannot be seen, as in the case of CFG (see micrographs E1 and E2 in Figure 2). The surface porosity is divided into micropores (pores of internal diameter < 2 nm), mesopores (pores of internal diameter between 2 nm and 50 nm), and macropores (pores of internal diameter > 50 nm) [82]. In this case, BO300S, BO500S, BO300L, BO500L, CFG, and CAC would be classified as modifiers with mesopores, which can be used for liquid–solid adsorption [83]. Nevertheless, this classification is considered referential and does not rule out the presence of micropores or macropores due to the abundance of pores of various sizes per sample and the variability among samples of the same material [84]. In BO, micropores are likely formed by gas release from the oat hull vesicles during pyrolysis. In contrast, mesopores and macropores could emerge from the vascular structure of the oat hull [83]. On the other hand, BO has a D_P_ similar to those of CFG and CAC but with a lower V_P_. This indicates that the BO pores are shallow and may reduce the amount of liquid or solid material that could be adsorbed during the asphalt binder modification procedure compared to the commercial modifier controls. 

Figure 2I,J show the particle size distribution of BO, CFG, and CAC obtained by laser diffraction. These distributions indicate that most of the particles fulfill the established particle sizes (<20 µm and <75 µm, respectively), indicating that the grinding performed meets the requirements of the study. Particles exceeding these sizes ranged from 1% to 7% in the BO500S, BO300L, BO500L, CFG, and CAC samples. In BO300S, however, this volume amounts to ~37%. This may be due to the presence of narrow and elongated particles that could pass through the mesh section of the sieve. Table 3 shows the descriptive parameters of the particle size distribution of BO and commercial modifier controls.

### 3.2. Physicochemical Characterization of BO and Commercial Modifier Controls

In this section, the results of the physicochemical properties of BO are analyzed to determine possible interaction mechanisms with the asphalt binder, which may explain its effect on its rheological properties. In this sense, the literature has shown that biochar is a highly carbonaceous material whose total carbon combines organic (C_org_) and inorganic carbon that can vary after pyrolysis [81]. C_org_ is generally stored in condensed aromatic rings with reactive functional groups that generate a high resistance to degradation [85]. It is fundamental to consider biochar an effective tool for C sequestration and CO_2_ emission reduction [81]. The SEM–EDX microelemental analysis provides a reference for the chemical composition of the BO and the commercial modifier controls used in this study (Table 4). From these data, it is inferred that BO is mostly composed of C and that this amount does not show significant differences when the oat hulls are pyrolyzed at 300 °C or 500 °C, according to the results obtained from Student’s *t* test (sig. value equal to 0.147). However, an upward trend is noted between the increase in PT and the amount of C in these samples, as described in the literature [79,81,86]. In that sense, the C content of the biochar can vary between 36% and 94%, depending on the raw material used and the PT. Therefore, the higher the PT, the higher the C content in the biochar [79,81,86]. Other inorganic elements, such as O, Mg, Si, P, K, and Ca, were also detected. O is the second predominant chemical element in BO, the amount of which decreases as the PT increases. This indicates that it could be transformed or eliminated with increasing temperature. On the other hand, the remaining chemical elements are present in similar amounts in all BO samples, with values below 5 wt% at both 300 °C and 500 °C. CFG and CAC are also primarily composed of C; however, according to the ANOVA, the amounts do not represent a significant difference compared to the amount of C in BO (*p*-value equal to 0.3999). On the other hand, CFG differs from BO and CAC due to the presence of some metallic compounds that were only found in its structure, corresponding to Ti, Cr, Fe, and Cu. Meanwhile, CAC presents a microelemental structure similar to that of BO, although with the presence of Al and a slight increase in the amount of Si.

**Figure 3:**
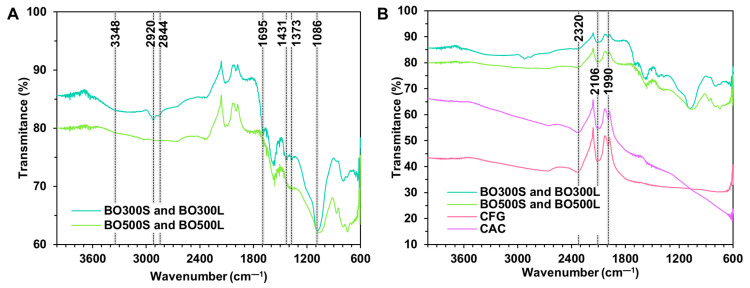
FTIR spectra. (**A**) compares the spectra of BO300S and BO300L to BO500S and BO500L. (**B**) compares the spectra of BO and the commercial modifier controls, CFG and CAC.

The FTIR analysis identified characteristic functional groups between BO pyrolyzed at 300 °C and 500 °C (Figure 3A) and between these and the commercial modifier controls (Figure 3B). The first difference observed in Figure 3A is in the 3348 cm^−1^ band, corresponding to the weak symmetric stretching of hydroxyl —OH. This only appears in the spectra of BO300S and BO300L, indicating that as the pyrolysis temperature increased, these groups could have been eliminated or transformed [33]. A similar situation is observed at 2920 cm^−1^ and 2844 cm^−1^, with the absence of the aliphatic groups —CH_3_ and —CH_2_ in the spectra of BO500S and BO500L. In this same spectrum, at 1695 cm^−1^, a decrease in the intensity of carbonyl —C=O bonds is observed until they practically disappear. The absence of these peaks and the previous ones is attributed to the decrease in some oat hull compounds, such as celluloses, hemicelluloses, ketones, and quinones [33,56]. These could have been transformed due to the reactions generated with the increase in PT for the production of BO. At 1431 cm^−1^ and 1373 cm^−1^ of the spectra of BO500S and BO500L, the broadening of the peaks corresponding to —C=C and —C–O is observed. These vibrations are attributed to the lignin in oat hulls, the peaks of which increase with PT. The stretching of the aliphatic ester —C–O is located at 1086 cm^−1^, which decreased in intensity in BO500S and BO500L due to the dehydration processes that developed as the pyrolysis temperature increased [87,88]. Concerning BO and the commercial modifier controls (CFG and CAC), the most evident difference is that BO is rich in functional groups (Figure 3B), showing more vibrations at wavenumbers between 1600 cm^−1^ and 700 cm^−1^ due to the composition of the oat hull [33]. The functional groups in this region usually have strong chemical bonds caused by the interaction between several functional groups. In addition, the vibrations located towards the lower wavenumbers (<900 cm^−1^) indicate the presence of aromatic rings [89]. On the other hand, BO300S and BO300L would have a higher surface acidity than the rest of the modifiers due to a higher number of peaks corresponding to carboxylic groups [33,90]. Finally, another difference between BO and the commercial modifiers is located at 2320 cm^−1^, 2106 cm^−1^, and 1990 cm^−1^ due to the drop in the intensity of the peaks corresponding to the alkyne bonds —C≡C in BO.

### 3.3. The Physicochemical Characterization and Storage Stability of the Modified Asphalt Binder

**Figure 4:**
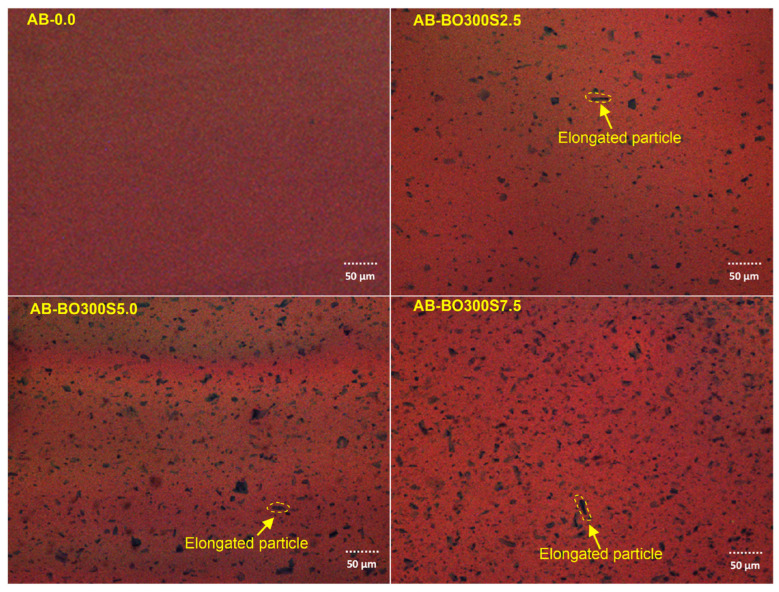
Confocal laser microscopy images showing the distribution of different percentages of BO300S in the asphalt binder. Yellow arrows indicate elongated BO300S particles.

The confocal laser microscopy images in Figure 4 were used to verify the distribution of different amounts of BO300S in the asphalt binder. These images show the BO300S particles in black; the rest correspond to the asphalt binder. After the modification process, the BO300S particles are generally homogeneously distributed in the asphalt binder for all the evaluated percentages (2.5%, 5%, and 7.5%, respectively). This indicates that the physical or consistency characteristics may remain similar for all regions of the sample. Some narrow and elongated particles in these images are consistent with the results in Figure 2I on the particle size distribution of BO300S. The distribution of the BO with a particle size <75 µm was evaluated in a previous study by Martínez-Toledo et al. [34]. According to the literature, similar behaviors have also been observed in other types of biochar, such as that from straw [91]. This type of biochar presents morphologically porous particles just like BO300S.

The characterization of the BO-modified asphalt binder also included performing an SEM–EDX microelemental analysis, the results of which are presented in Table 5. As shown in the microelemental analysis performed on BO (see Table 4), there may be fluctuations in the content of a chemical element depending on the region where the data capture is performed [90]. Nevertheless, this can provide essential information to understand the general chemical composition of the modifications. In this case, C, O, and S are the major components found in the samples analyzed, with C being the predominant chemical element. According to Student’s *t* test for normal data, no statistically significant differences were found between the mean of the AB and AB-0.0 components, with a sig. value equal to 0.143, 0.450, and 0.086 for C, O, and S, respectively. This indicates that the modification procedure alone would not generate significant chemical changes in the asphalt binder. In the case of asphalt binders modified with the same amount of BO (5%), S is contributed by the asphalt binder since it is a compound that belongs neither to oat hulls nor to BO [33,34,56]. Regarding C and O, the ANOVA indicated that no statistically significant differences were found between the mean of the asphalt binders modified with BO and the AB, considering a significance level of 95% confidence (*p*-value of 0.0540 and 0.05214 for C and O, respectively).

**Figure 5:**
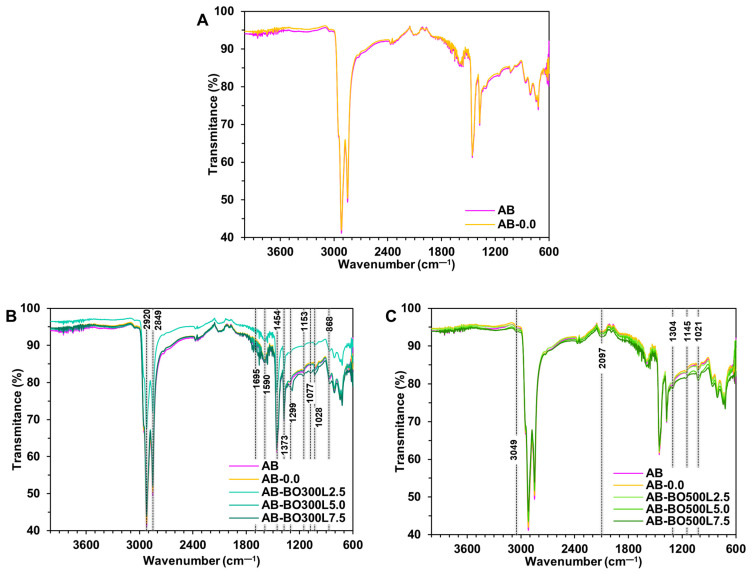
FTIR spectra. (**A**) comparison between AB and AB-0.0. (**B**) comparison between AB, AB-0.0, and different percentages of AB-BO300L. (**C**) comparison between AB, AB-0.0, and various percentages of AB-BO500L.

FTIR spectra were also obtained for some samples to identify characteristic functional groups after the modification of the asphalt binder. As illustrated in Figure 5A, the spectra of the AB and AB-0.0 present similar bond intensities and vibrations within the range evaluated (between 4000 cm^−1^ and 600 cm^−1^). This indicates that the modification procedure alone would not generate variations in the functional groups of the samples. Therefore, the differences observed in the rest of the spectra could be attributed to interactions between the asphalt binder and the BO. AB-BO300L (Figure 5B) shows that the BO could be reacting with the asphalt binder through various functional groups. Hence, vibrations with different intensities are observed depending on the amount of BO300L added to the asphalt binder. Generally, with 5% and 7.5% BO300L, bond vibrations are more intense than with 2.5%. This can be observed in different bands, such as 2920 cm^−1^ and 2849 cm^−1^ due to the strong symmetric stretching of —CH_3_ and —CH_2_, respectively; 1695 cm^−1^ due to —C=O stretching; 1590 cm^−1^ and 1454 cm^−1^ corresponding to the stretching of the alkene —C=C with weak and strong intensity, respectively; 1373 cm^−1^, 1153 cm^−1^, and 1077 cm^−1^ due to —C–O stretching; 1299 cm^−1^ corresponding to weak —C–H stretching, with a shift to a lower energy value (1282 cm^−1^) when using 7.5% BO300L; 1028 cm^−1^ due to —C–C stretching; and at 868 cm^−1^ due to —C–O–C stretching. In AB-BO500L (Figure 5C), characteristic bond vibrations are also observed, and their intensities are proportional to the amount of BO500L added to the asphalt binder. The following can be observed: 3049 cm^−1^ with the weak symmetric stretching of —CH_3_; 2097 cm^−1^ corresponding to the stretching of the alkyne —C≡C; 1304 cm^−1^ as a result of the stretching of —C–H; 1145 cm^−1^ corresponding to —C–O; and at 1021 cm^−1^ corresponding to —C–C. The above peaks are indications of possible interactions between BO300L and BO500L with the asphalt binder.

**Figure 6:**
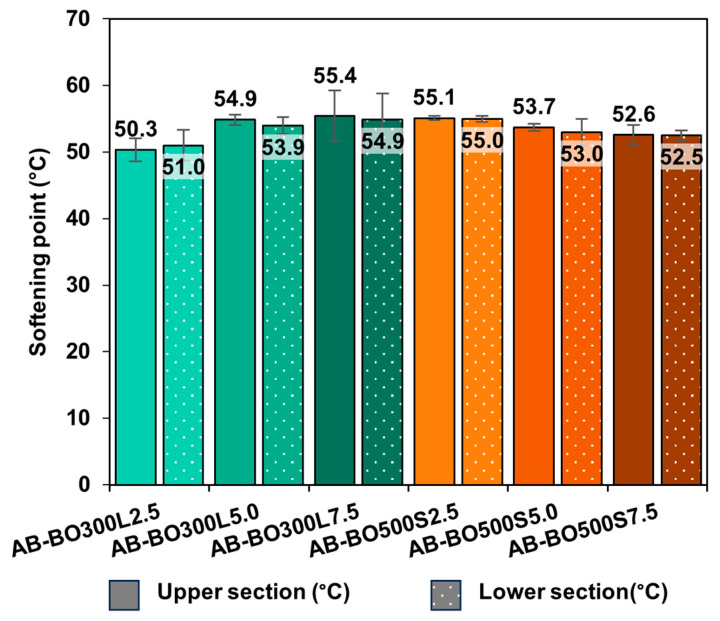
Storage stability of AB-BO300L and AB-BO500S with different modification percentages.

As shown in the confocal laser microscopy images (see Figure 4), BO does not dissolve in the asphalt binder. For this reason, two phases can be distinguished within the same sample: one corresponding to BO and the other to the asphalt binder. The compatibility of these phases was studied through the storage stability test; the results appear in Figure 6. This figure shows the numerical difference between the softening points of the upper and lower sections of AB-BO300L and AB-BO500S, including different modification percentages (2.5%, 5%, and 7.5%, respectively). In this regard, the results show that the compatibility of the system is stable in all the samples evaluated because the difference between the softening points of the upper and lower sections is less than 2.5 °C [34,92,93]. Moreover, the ANOVA (*p*-value equal to 0.0607) shows no statistically significant differences. This good storage stability may be due to the porous structure of BO, which gives it a low density at both <20 µm and <75 µm particle sizes. This makes it easier for them not to accumulate at the bottom of the containers, unlike other asphalt binder modification materials, such as mineral filler and graphite (2.699 g/cm^3^ and 2.1 g/cm^3^, respectively) [59]. In addition, the strong interactions produced between the BO and the asphalt binder through chemical bonds (see Figure 5B) could cause BO particles to tend to maintain their position within the asphalt binder.

### 3.4. Evaluation of Effects of PT, Particle Size, and Amount of BO at High and Low Temperatures on Asphalt Binder

#### 3.4.1. High Temperature Evaluation Using the Rutting Parameter G*/sin(δ)

**Figure 7:**
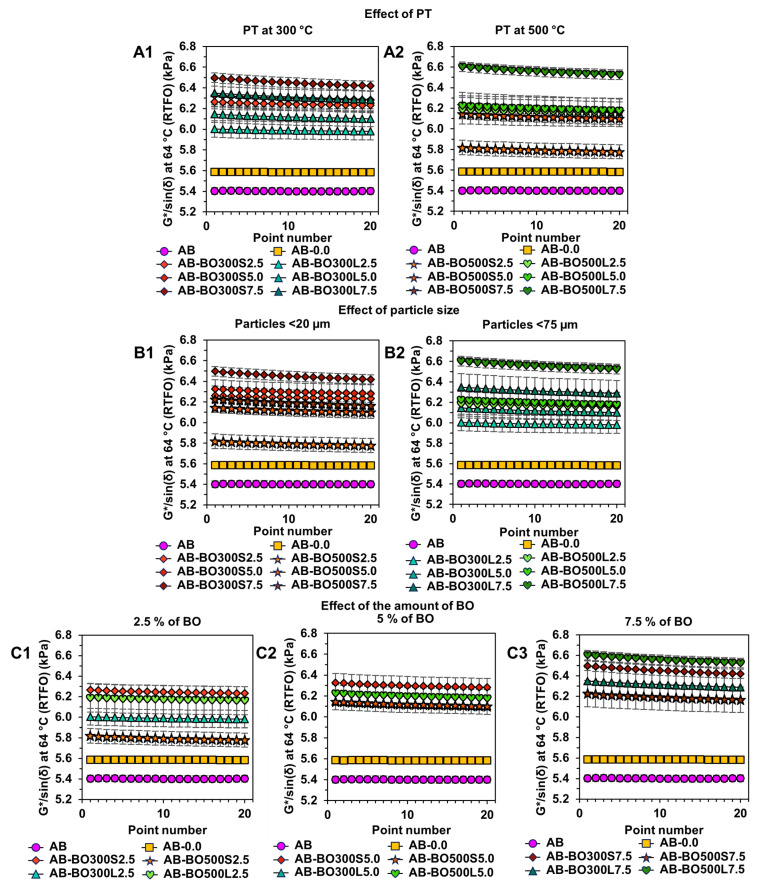
The effect of BO factors on the rutting parameter G*/sin(δ) of the asphalt binder evaluated at 64 °C previously subjected to short-term aging in the RTFO. (**A1**,**A2**) correspond to the PT effect of 300 °C and 500 °C, respectively. (**B1**,**B2**) correspond to the effect of particle size <20 µm and <75 µm, respectively. (**C1**–**C3**) correspond to the effect of the amount of BO equivalent to 2.5%, 5%, and 7.5%, respectively.

Figure 7 shows the effects of BO on the rutting parameter G*/sin(δ) of asphalt binders evaluated at 64 °C, previously subjected to short-term aging in the RTFO. The R^2^ of the factorial model was higher than the R^2^ of the initial model, indicating a good fit of the results for evaluating the rutting parameter G*/sin(δ) (99.68% and 88.44%, respectively).

Graphs A1 and A2 in Figure 7 show that the PT of the BO had a positive effect on the rutting parameter of the asphalt binder, becoming one of the main significant factors for improving the resistance of the asphalt binder at high service temperatures (*p*-value equal to 0.029 in ANOVA). According to these results, the effect of the PT was more evident with a 7.5% BO. For example, at a PT of 300 °C, the improvements in the rutting parameter ranged from about 10% to 16%, with AB-BO300S7.5 being the sample with the highest deformation resistance compared to AB. However, at a PT of 500 °C, these improvements were between 7% and 18%, with AB-BO500L7.5 being the most resistant.

When evaluating the effect of BO particle size (see graphs B1 and B2 in Figure 7), the ANOVA indicated that the individual impact of this factor does not represent a significant difference in the rutting parameter of the asphalt binder (*p*-value equal to 0.052). However, particle size is a factor that depends on the PT used. This implies that the combinations generated between the levels of these factors are capable of causing a statistically significant effect on the rutting parameter (*p*-value equal to 0.004, considering a 95% confidence level). Consequently, to obtain a greater resistance of the BO-modified asphalt binder, it is preferable to use particle sizes <20 µm with a PT of 300 °C or particle sizes <75 µm with a PT of 500 °C. In the former case, more particles per area of the asphalt binder would be capable of interacting with it due to strong chemical bonds caused by the functional groups of both materials (see Figure 3A and Figure 5). In the second case, however, the benefits would be through physical interactions, given by the microscopic morphology of the pyrolyzed BO at 500 °C due to these temperatures causing highly porous particles with a higher S_BET_ than the other BO (see Figure 2G).

The amount of BO also corresponds to one of the main significant factors for improving the rutting parameter (*p*-value equal to 0.007 in the ANOVA). As shown in graphs C1 and C2 of Figure 7, this effect is proportional to the amount used to modify the asphalt binder. In cases where 2.5%, 5%, and 7.5% BO were used, the rutting parameter increased by up to 13%, 14%, and 18%, respectively, compared to AB. In addition, the AB-BO300S and AB-BO500L samples showed the best performances at high temperatures, coinciding with the results obtained in the PT and particle size analyses. In general, both samples are composed of porous and irregular particles that could help generate a better interaction with the asphalt binder, increasing its stiffness [8,9,11,68,69]. In addition, their FTIR spectra present several peaks attributed to the vibrations produced between the chemical bonds of the asphalt binder and the BO (see Figure 5), which are usually strong due to the interactions among several functional groups [89].

#### 3.4.2. An Evaluation at Low Temperatures Using the Fraass Breaking Point

The BO factors regarding the Fraass breaking point of asphalt binders were also evaluated using the 2 × 2 × 3 full factorial design. In this case, however, the R^2^ was equal to 99.65%, and the predicted R^2^ was 87.40%, indicating a good fit of the results. 

**Figure 8:**
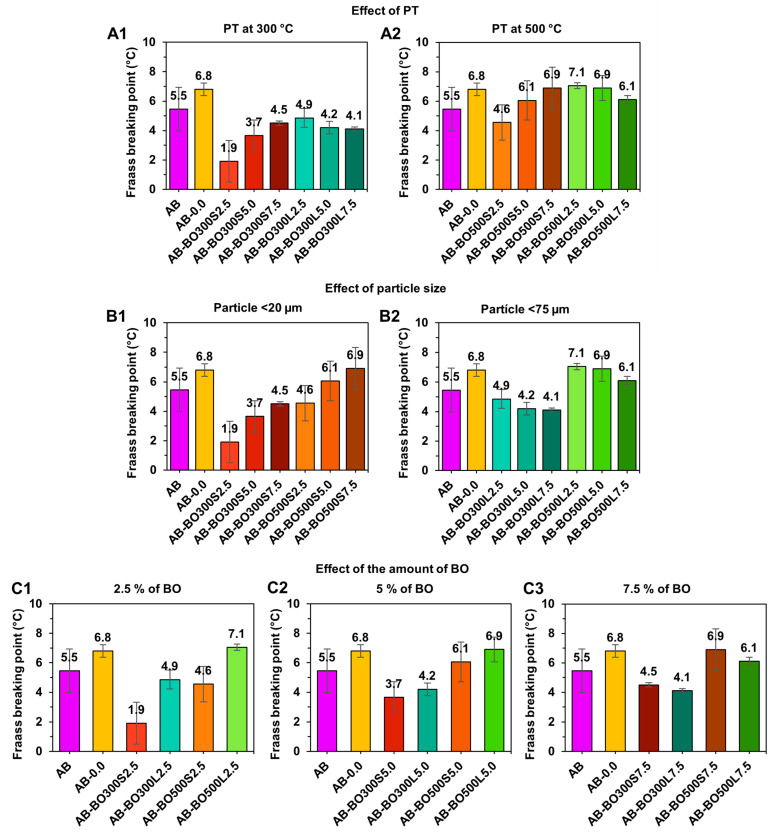
The effect of BO factors on the Fraass breaking point of the asphalt binder with long-term aging in a PAV. (**A1**,**A2**) correspond to the PT effect of 300 °C and 500 °C, respectively. (**B1**,**B2**) correspond to the effect of particle size <20 µm and <75 µm, respectively. (**C1**–**C3**) correspond to the effect of 2.5%, 5%, and 7.5% of BO, respectively.

From Figure 8, the ANOVA indicated that the PT and particle size of the BO are the main factors causing significant effects on the Fraass breaking point of the asphalt binders (with *p*-values equal to 0.003 and 0.018, respectively). In contrast, the factor of the amount of BO does not represent a significant difference, i.e., by itself, it was not able to reduce the Fraass breaking point of the modified asphalt binders compared to AB (with a *p*-value equal to 0.067 from the ANOVA). However, when combined with certain particle sizes, this factor significantly improved sample performance (*p*-value equal to 0.016 of the ANOVA).

To perform a multiple comparison to determine which means were significantly different from the Fraass breaking point of AB, Fisher’s least significant difference (LSD) test was performed. This indicated that, for a PT of 300 °C (see graph A1 in Figure 8), only AB-BO300S2.5 significantly reduced the Fraass breaking point equivalent to ~4 °C. The rest of the samples showed no statistical differences. On the other hand, at a PT of 500 °C (see graph A2 in Figure 8), no significant improvement compared to AB was observed. According to the particle size results (see graphs B1 and B2 in Figure 8), several samples showed tendencies to reduce the breaking temperature of the asphalt binders. However, only particle sizes <20 µm reached a significant effect, where sample AB-BO300S2.5 stood out, coinciding with the previous analysis. Regarding the effect of the amount of BO (see graphs C1 and C2 in Figure 8), the results indicate that dosages of 2.5% combined with particle sizes <20 µm are preferable to achieve the lowest cracking temperatures.

According to the literature, using biochar to modify asphalt binders involves controlling the amount of modifier added and the particle size [10,22]. This is because these variables could increase the critical stiffness of the asphalt binder, making it more prone to cracking [1]. In this case, the Fraass breaking points obtained were similar to that of AB and, in some cases, even improved (AB-BO300S2.5). This indicates that BO would not detrimentally impact asphalt binders at low service temperatures.

#### 3.4.3. Sample Selection According to the Increase in Viscoelastic Range

The viscoelastic range of the asphalt binder is limited by the viscous and solid elastic behavior, which depends on temperature and loading time [1]. The viscoelastic range defines the resistance to the rutting and thermal cracking of asphalt pavements depending on the increase or decrease in its components: viscous (non-recoverable) and elastic (recoverable). In addition, it corresponds to the best working condition for an asphalt binder, since it allows it to dissipate stress in the form of deformation. This could be recovered as a function of time once the load is removed [4,5].

**Figure 9:**
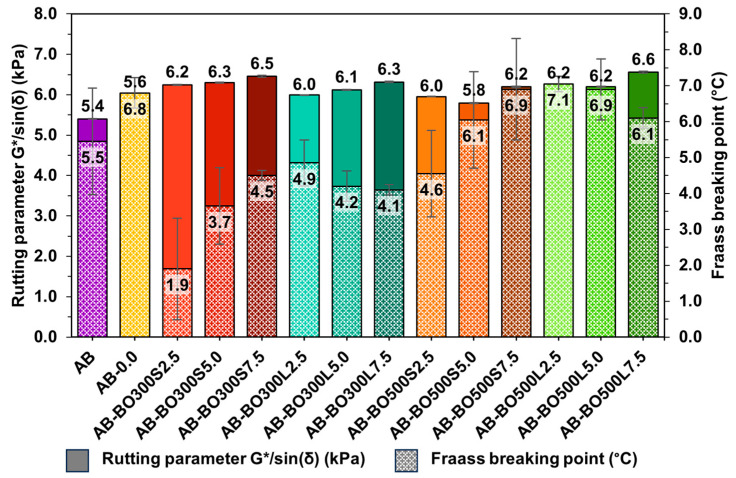
Viscoelastic range as a function of the rutting parameter G*/sin(δ) and the Fraas breaking point.

Based on the results of the 2 × 2 × 3 full factorial design on the rutting parameter G*/sin(δ) and the Fraass breaking point, the asphalt binders with BO that tended to extend their viscoelastic range the most were AB-BO300S2.5, AB-BO300S5.0, and AB-BO300S7.5 (Figure 9), showing an increase in the rutting parameter around ~16% (for AB-BO300S7.5) and a decrease in the Fraass breaking point of around ~4 °C (for AB-BO300S2.5), compared to AB. AB-BO300S5.0 maintained an intermediate improvement between AB-BO300S2.5 and AB-BO300S7.5. Thus, the samples selected for a further evaluation of their rheological properties were AB-BO300S2.5, AB-BO300S5.0, and AB-BO300S7.5.

### 3.5. Evaluation of Rheological Properties of Modified Asphalt Binder

#### 3.5.1. Flow Resistance and Workability

**Figure 10:**
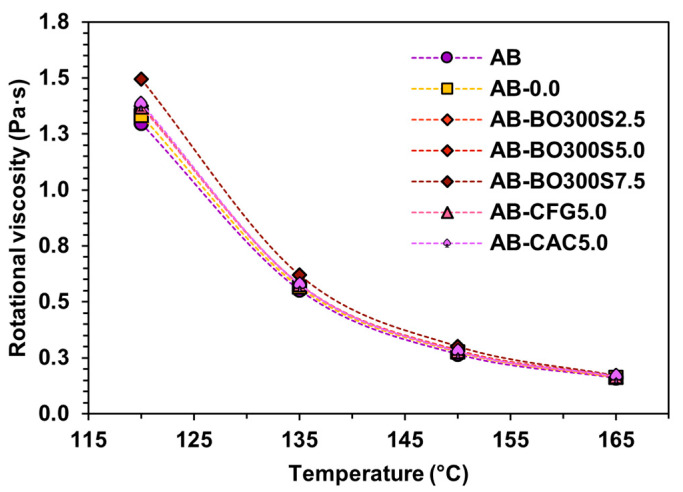
Rotational viscosity at 120 °C, 135 °C, 150 °C, and 165 °C in the original condition of the samples.

Figure 10 presents the rotational viscosity obtained between 120 °C and 165 °C in samples evaluated in the original state to determine flow resistance and workability. First, all samples tended to exceed the flow resistance and workability of AB, which is reflected in the increased rotational viscosity in the temperature range evaluated. This behavior was also noted in AB-0.0, which showed a rotational viscosity between 3% and 6% higher than AB, with significant differences between them (*p*-value equal to 0.000 between 120 °C and 150 °C and *p*-value equal to 0.035 at 165 °C in the ANOVA). This may happen due to chemical reactions generated by the organic molecules of the asphalt binder based on the temperature used during the modification procedure (160 °C for 30 min) [11,94]. This temperature could cause the molecules to polymerize, forming asphaltenes and aromatics, i.e., the dispersion medium of asphaltenes to be reduced, increasing the rotational viscosity of the asphalt binder [95]. On the other hand, the samples with BO300S also presented a higher flow resistance and workability than AB and even AB-0.0. In addition, they obtained significant differences in the mean rotational viscosity of these samples for all temperatures evaluated according to the ANOVA and Fisher’s LSD (*p*-value equal to 0.000, considering a 95% confidence level). In that sense, 120 °C is when the greatest differences appear; for example, adding 2.5% and 5% BO300S, both samples increased their flow resistance and workability by ~6% and ~2% compared to AB and AB-0.0, respectively. However, 7.5% BO300S increased by ~13% and ~9% compared to the same control samples. Accordingly, the results indicate that the increase in the flow resistance and workability of the asphalt binders evaluated is due to both the effect of the modification procedure and the addition of BO300S. 

On the other hand, adding 2.5% and 5% BO300S had a similar effect to that caused by commercial modifier controls. However, with 7.5% BO300S, the flow resistance and workability were higher. Additionally, 7.5% BO300S increased the rotational viscosity by ~11% on average compared to AB. With 5% CFG or CAC, this only increased by ~3% or ~6% (on average), respectively. This indicates that the increase in the flow resistance and workability of the asphalt binder may also be due to the type and amount of modifier used. In this regard, a greater number of particles compatible with the asphalt binder could generate more physicochemical interactions among them, improving its internal matrix and, therefore, its rotational viscosity [8,11,22,30]. Thus, the higher the BO300S content, the higher the flow resistance and workability of the asphalt binder. Additionally, this could aid in improving the rutting resistance of the asphalt binder at high service temperatures in a pavement [8,9]. On the other hand, the results obtained indicate that BO300S and the commercial modifier controls would fulfill the Superpave specification on obtaining a rotational viscosity of less than 3 Pa·s at 135 °C.

The increase in rotational viscosity is usually associated with an increase in the temperature required to mix and compact the hot mix asphalt (HMA) because the modified asphalt binder would present a higher flow resistance and workability than a conventional asphalt binder [1]. On this basis, the mixing and compaction temperatures of the samples studied here were determined as a function of the rotational viscosity obtained at 2 poises and 3 poises, respectively. According to the results shown in Table 6, using 7.5% BO300S would require increasing the mixing and compaction temperatures by ~3 °C compared to AB. The remaining samples would require slightly higher temperatures (between 1 °C and 2 °C, approximately). These results agree with previous research by Martínez-Toledo et al. [34], who evaluated the rotational viscosity of asphalt binders modified with 2.5%, 5%, and 7.5% BO with a particle size <75 µm, concluding that these asphalt binders also required increased mixing and compaction temperatures but between 5 °C and 7 °C, respectively. This is due to an increase in rotational viscosity, which was higher than that obtained by the samples in this study.

#### 3.5.2. High Temperature Performance

**Figure 11:**
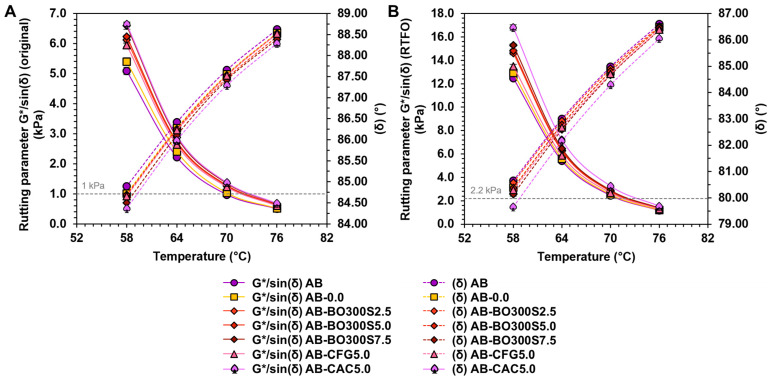
Rutting parameter G*/sin(δ) versus phase shift angle (δ) obtained between 58 °C and 76 °C in asphalt binders: (**A**) in the original state, and (**B**) short-term aged in the RTFO. The gray dashed lines indicate the allowable Superpave specification for the rutting parameter G*/sin(δ).

Figure 11A shows the results of the rutting parameter G*/sin(δ) and phase shift angle (δ) of asphalt binders evaluated in the original state to determine their rutting resistance. Based on these results, the samples with BO300S improved the performance of the asphalt binder at high temperatures, presenting a higher rutting parameter than AB and AB-0.0. On average, the samples with 2.5%, 5%, and 7.5% BO300S had a rutting parameter ~19%, ~21%, and ~25% higher than AB, respectively. When comparing them to AB-0.0, they obtained a rutting parameter between ~17% and ~24% higher. These results showed significant differences compared to the mean of AB and AB-0.0 according to the ANOVA (*p*-value equal to 0.000) and Fisher’s LSD (considering a 95% confidence level). This indicates that BO300S could positively affect the rutting resistance of asphalt binders, with the increase in the rutting parameter being directly proportional to the amount of modifier used. As seen in Figure 11A, BO300S tends to decrease the phase shift angle (δ) of the asphalt binder, causing an increase in its elastic component. This effect would improve the performance of the asphalt binder at high service temperatures, allowing it to act as a more resistant material against permanent deformations due to its improved elastic response [8,9]. Further analysis shows that all asphalt binders with BO300S achieved better performance at a high temperature than the asphalt binder modified with CFG, even for the same amount of modifier. For example, 5% BO300S resulted in a 4% higher rutting parameter than 5% CFG. This could be attributed to some CFG particles having smooth and dense surfaces, unlike BO300S, which has mainly porous particles that could function as a resistant skeleton within the asphalt binder, causing a stiffening effect [9] (see SEM micrographs A1, A2, E1, and E2 in Figure 2). Moreover, this effect coincides with the results obtained regarding the flow resistance of the modified asphalt binder (see Figure 10) and with other studies on the use of biochar with characteristics similar to those of this study [8,11,22,30]. Regarding the application of 5% CAC, this showed improvements in high temperature performance similar to BO300S and CFG, reaching a rutting parameter ~5% and ~9% higher than that caused by the same amount of BO300S and CFG, respectively. In addition, it obtained the lowest phase shift angle (δ) compared to the rest of the samples. This may be due to its highly porous and rough particles, which have practically no smooth surfaces, resulting in better internal adhesion with the asphalt binder [61,64,65]. These results align with other studies in which environmentally friendly materials are used to enhance the rheological properties of compounds or biocomposites applicable in the field of civil engineering [96].

As shown in Figure 11B, after short-term aging in the RTFO, all the samples had a higher rutting resistance, reaching a rutting parameter G*/sin(δ) higher than that obtained in their original state (Figure 11A) due to the oxidation of the asphalt binder [22]. The samples with BO300S also had a higher rutting parameter than the control samples (AB and AB-0.0), which increased directly proportionally to the amount of BO300S added to the asphalt binder. According to the results, the applications between 2.5% and 7.5% BO300S increased rutting resistance, reflected in the increase in the rutting parameter between ~15% and ~19% on average compared to AB. Conversely, compared to AB-0.0, these increased between ~11% and ~16% on average. In both cases, significant differences were obtained compared to the mean of the control samples (AB and AB-0.0) and between the samples with different amounts of BO300S to modify the asphalt binder, according to the ANOVA (*p*-value equal to 0.000) and Fisher’s LSD. In addition, all the samples with BO300S tended to reduce their phase shift angle (δ), which could be beneficial for improving the strength of the asphalt binder at high service temperatures [8,9] due to the increased elastic response of the material. Compared to commercial modifier controls, using BO300S would enable intermediate performance between CFG and CAC to modify the asphalt binder. This is because, when used at the same amount (5%), BO300S achieves a phase shift angle (δ) similar to CFG but with a ~7% higher rutting parameter. However, BO300S obtains a phase shift angle (δ) with a smaller elastic component and a ~17% lower rutting parameter compared to CAC. In other words, this indicates that the CAC-modified asphalt binder would perform the best at high service temperatures, acting as a more elastic and rutting-resistant material. However, part of this good performance could also be due to a higher polymerization of its organic molecules due to the action of the temperature, indicating that it would be a material more susceptible to aging than the other modifiers [11,94,95]. With AB-0.0, the situation is similar, since this sample presents greater stiffening than AB due to an increase in its rutting parameter G*/sin(δ). This is attributed to aging caused by modification.

Based on the results of BO300S-modified asphalt binders, they could fulfill the Superpave specification at the high PG temperature of AB (64 °C) by obtaining rutting parameters G*/sin(δ) over 1 kPa and 2.2 kPa in the original and the RTFO-aged state, respectively. However, the critical temperatures would also increase by approximately 3 °C to 4 °C depending on the amount of BO300S used.

**Figure 12:**
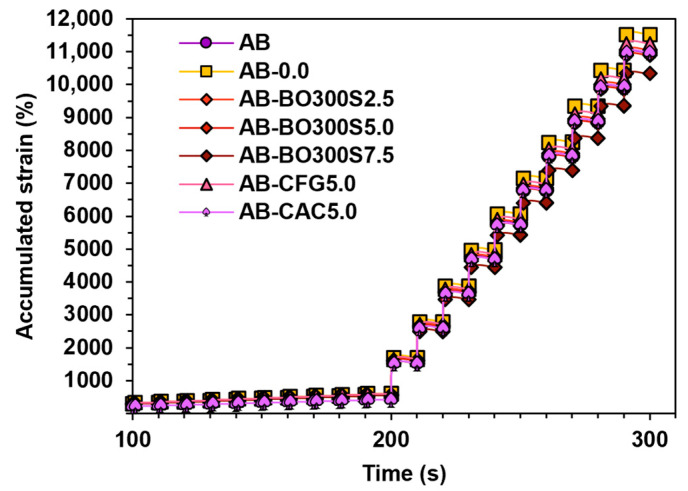
Cumulative deformation obtained at 70 °C according to the MSCR test. Up to 200 s, a stress level of 0.1 kPa was applied, and between 200 s and 300 s, a stress level of 3.2 kPa was applied.

Figure 12 shows the cumulative deformation results under different stress levels at 70 °C. Up to 200 s, the first 10 loading cycles were evaluated at a stress level of 0.1 kPa. The next 10 loading cycles were evaluated between 200 s and 300 s with a stress level of 3.2 kPa. After the completion of the test, the samples with 2.5% and 5% BO300S obtained a cumulative deformation similar to that of AB. However, with 7.5% BO300S, this decreased by about 6%, obtaining a significant difference compared to the mean AB according to Fisher’s LSD (test statistic equal to 1126.22). Compared to the commercial modifier controls, using 5% BO300S caused a deformation similar to that obtained by 5% CFG or CAC. Furthermore, according to the ANOVA test, these samples did not show a significant decrease compared to the AB mean (*p*-value equal to 0.8148). This indicates that BO300S would have to be used at a minimum amount of 7.5% to affect the cumulative permanent deformations of the asphalt binder positively. Higher percentages could be considered, since other authors have studied the effect of biochar from Mesua Ferrea seed cover, indicating that, at amounts higher than 10% biochar, the cumulative deformations would be lower than those obtained by a conventional asphalt binder [68].

**Figure 13:**
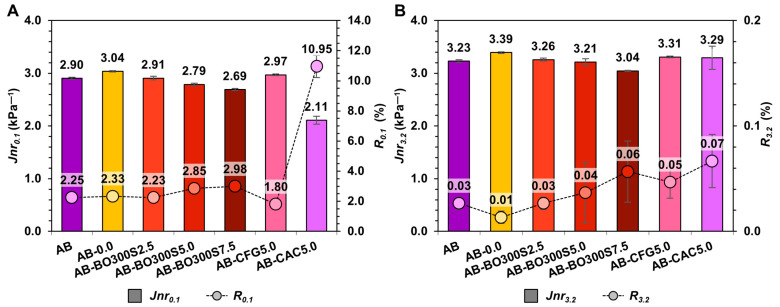
Non-recoverable creep compliance (Jnr) versus recovery percentage (R%) under different stress levels: (**A**) 0.1 kPa and (**B**) 3.2 kPa.

Figure 13A,B show the non-recoverable creep compliance (Jnr) recovery percentage (R%) under different stress levels obtained using the MSCR test (0.1 kPa and 3.2 kPa, respectively). Regarding the Jnr parameter, the lower its value, the higher the resistance of the asphalt binder to rutting due to a lower residual deformation after a creep cycle [64,68]. In this sense, the results indicate that the application of BO300S tends to increase rutting resistance, which is reflected in the tendency to reduce Jnr for both stress levels (Figure 13A,B), being proportional to the amount added to the asphalt binder. In this case, the sample with 7.5% BO300S achieved a statistically significant improvement in rutting resistance compared to AB, decreasing Jnr by ~8% and ~6% at 0.1 kPa and 3.2 kPa, respectively (with a sig. value equal to 0.039 and 0.001 in the Bonferroni test). In the case of asphalt binders with the commercial modifier controls, the sample with 5% CAC had the best rutting resistance, since it obtained the highest Jnr at low stress levels (less than ~37% compared to AB). However, with the increased applied load, this condition was lost, behaving like the rest of the modifiers, either with 2.5% or 5% BO300S, or 5% CFG. This indicates that the combination of particle sizes <20 µm and modification percentages of 7.5% BO300S would have a better effect on the permanent deformation resistance of the asphalt binder [64,65]. This coincides with the results obtained for the rutting parameter G*/sin(δ) analyzed in Figure 11A,B.

When analyzing R%, the increase in BO300S in the asphalt binder tends to increase R% for both stress levels. However, according to the Bonferroni pairwise comparison test of groups, they do not achieve significant differences compared to AB. For example, for a stress level of 0.1 kPa, the asphalt binders with 2.5%, 5%, and 7.5% BO300S obtained a sig. value between 0.086 and 1.000 compared to AB. At 3.2 kPa, these obtained a sig. value between 0.116 and 1.000. Compared to the commercial modifier controls, at a stress level of 0.1 kPa, the application of 5% BO300S had a slightly higher R% (~1%) than 5% CFG (with a sig. value equal to 0.040 in the Bonferroni test). However, the sample with 5% CAC achieved the highest R% compared to AB and the rest of the samples with the same modification percentage. For example, for the case of 5% BO300S, the CAC exceeded its R% by ~8%. On the other hand, at a stress level of 3.2 kPa, the R% of the samples with 5% BO300S, CFG, and CAC were equal to that obtained by AB. There were no significant differences between them according to the Kruskal–Wallis test with a sig. value equal to 0.174. This indicates that the modifiers used would maintain the elastic recovery capacity of AB at a stress level of 3.2 kPa. Therefore, at high service temperatures, they would not contribute new energy to re-establish the original shape of the asphalt binder after deformation [1,64,97]. 

Based on the results of Jnr_3.2_ and R%_3.2_ (Figure 13B), BO300S would not have the effect of elastomeric-type modifiers since it would not generate high recovery levels or a notable elastic response in the asphalt binder compared to a polymer.

#### 3.5.3. Performance at Intermediate Temperatures

**Figure 14:**
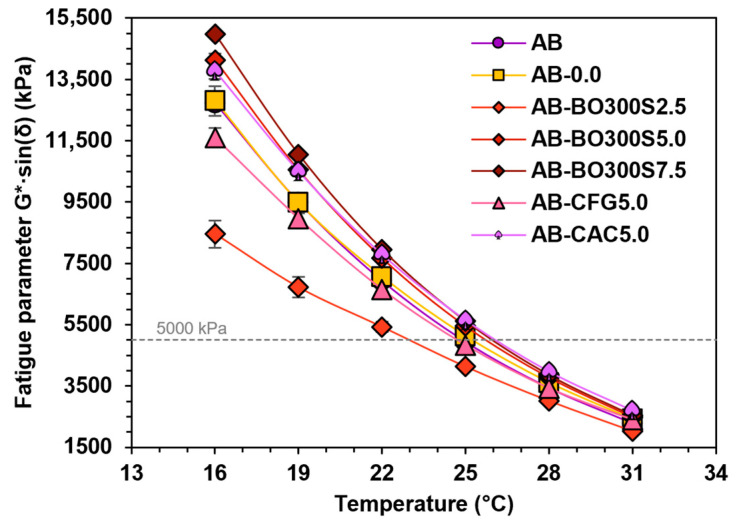
Fatigue parameter G*·sin(δ) evaluated between 16 °C and 31 °C in long-term aged samples by the PAV. The gray line represents the Superpave specification requirement for the fatigue parameter.

The performance of the asphalt binder at intermediate temperatures was evaluated using the fatigue parameter G*·sin(δ) on long-term aged samples by the PAV. According to the results shown in Figure 14, the application of 2.5% BO300S and 5% CFG would improve fatigue damage resistance, as they tended to decrease the fatigue parameter compared to AB. In the case of BO300S, this decrease was between ~11% and ~34% for the temperature range evaluated. With CFG, it ranged from ~2% to ~9% at temperatures between 16 °C and 25 °C (with no significant difference at higher temperatures, according to Fisher’s LSD). On the other hand, using 5% and 7.5% BO300S and 5% CAC would have an unfavorable effect on fatigue damage resistance because they would increase the fatigue parameter considerably. However, these results indicate the maximum amount that could be used to modify asphalt binders without affecting their performance at intermediate temperatures. According to the literature, the use of BO300S would have more favorable effects on the fatigue resistance of asphalt binders than those observed with other biochars of a larger particle size (<75 µm) [9,68]. However, to achieve such an effect, it is necessary to use modification percentages close to 2.5% as well as a particle size smaller than 20 µm. Otherwise, BO300S could significantly increase the stiffness of the asphalt binder due to its high content of functional groups (see Figure 3B), which exhibit binding vibrations between 1600 cm^−1^ and 700 cm^−1^ that are usually strong due to the interaction between various functional groups in the oat hull [33].

According to the Superpave specification, asphalt binders modified with 2.5% BO300S would meet the fatigue parameter of less than 5000 kPa at the intermediate service temperature (25 °C for the PG 64-22 asphalt binder). With 5% and 7.5% BO300S, this requirement could be fulfilled at temperatures between 25 °C and 28 °C.

#### 3.5.4. Low Temperature Performance

**Figure 15:**
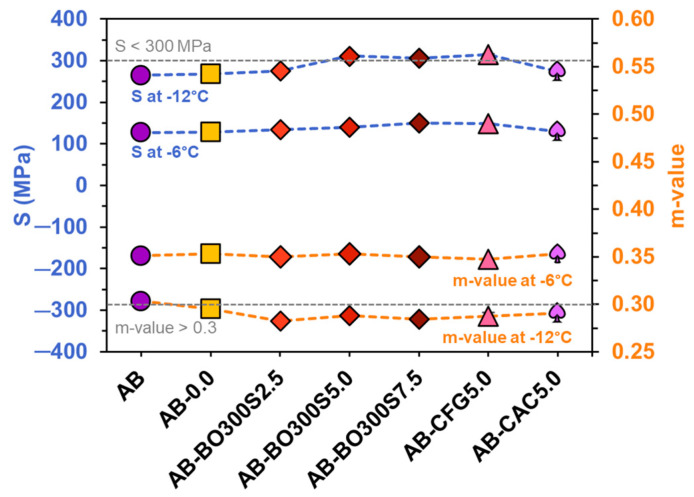
Creep stiffness (S) and m-value results at −6 °C and −12 °C obtained using the BBR test. The gray lines represent the Superpave specification requirement for the S parameter and m-value.

The BBR test was used to evaluate the performance of asphalt binders at low service temperatures, using long-term samples by the PAV. Figure 15 shows the results of the BBR test performed at −6 °C and −12 °C, where an increase in creep stiffness (S) and a decrease in m-value with a decreasing test temperature are observed. At −6 °C, both the asphalt binders with BO300S and those with the commercial modifier controls obtained an S-parameter and m-value similar to AB and AB-0.0, with no significant differences between them, according to the Kruskal–Wallis test with a sig. value equal to 0.056 and 0.095 for S and m-value, respectively. Despite not showing statistical differences between them, at −12 °C, some samples tended to increase the S-parameter and m-value compared to AB, in particular, those with the highest BO300S contents (obtaining a sig. value equal to 0.057 and 0.064 for S and m-value, respectively, according to the Kruskal–Wallis test). This could indicate that the BO300S-modified asphalt binder tends to exhibit greater strength and deformation (or flexibility) at low service temperatures. Based on this, the literature suggests that using biochar to modify an asphalt binder involves controlling its quantity and particle size because these factors could increase the critical stiffness of the asphalt binder, making it more prone to cracking [10,22]. In this case, the results showed that using BO300S would not impair the strength of the asphalt binder at low service temperatures, coinciding with the evaluation of the Fraass breaking point (see Figure 8). Therefore, BO300S could be used either at 2.5%, 5%, or 7.5% with respect to the weight of the asphalt binder for modification.

### 3.6. Evaluation of Susceptibility to Aging of Modified Asphalt Binder

**Figure 16:**
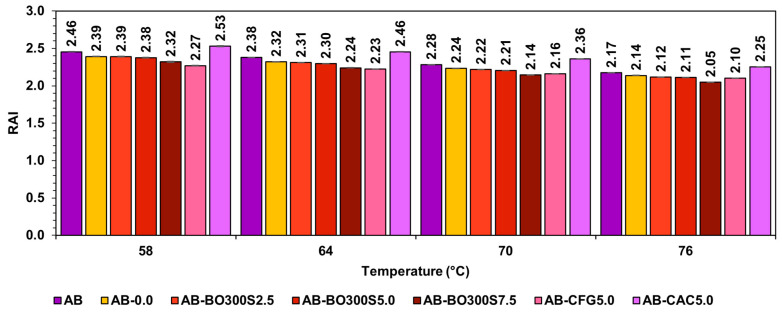
Rheological aging index (RAI) between 58 °C and 76 °C.

The susceptibility of asphalt binders to aging was evaluated by the rheological aging index (RAI) based on the results of the rutting parameter G*/sin(δ) in the original state and short-term aged in the RTFO. The results presented in Figure 16 indicate that the RAI tends to decrease as the amount of BO300S increases. For example, the use of 2.5%, 5%, and 7.5% BO300S caused the RAI to decrease by ~2%, ~3%, and ~6% on average compared to the RAI of AB, obtaining significant differences between them (*p*-value equal to 0.000 in the ANOVA, considering a 95% confidence level for all the temperatures evaluated). Comparing these results with those obtained by the commercial modifier controls, the sample with 5% BO300S had a ~3% higher RAI than the sample with 5% CFG. However, compared to the sample with 5% CAC, it achieved a ~6% lower RAI. The decrease in the RAI of the asphalt binders could indicate that the modifier is causing an improvement in the aging resistance [76]. In this regard, the FTIR analysis performed on BO300S (see Figure 3A) showed the presence of phenolic groups from the lignin of the oat hulls. It is composed of benzene rings linked to –OH groups that act as antioxidants, since their structures can neutralize oxygen-bearing free radicals, such as ketones and sulfoxides generated during the oxidation of the asphalt binder [68,98,99]. This effect could explain the decrease in the RAI in the presence of BO300S. On the other hand, Zhang et al. [22] state that stiffer asphalt binders tend to age less due to the effects caused by the biochar content. However, this stiffening effect could also be caused by the modification procedure used, since exposure to high temperatures (160 °C, in this case) could generate a higher polymerization of the organic molecules of the asphalt binder, increasing its viscosity and, therefore, its stiffness [11,94,95]. This could explain why the RAI of AB-0.0 is lower than that of AB at the different temperatures evaluated.

## 4. Conclusions

In this study, the effect of BO on the rheological properties of the asphalt binder at different service temperatures was evaluated using various material characterization techniques, physicochemical analyses, and rheological tests, which led to the following conclusions:

(a) BO production achieves similar yields for the pyrolysis treatment temperatures used. However, a more porous BO with a larger surface area can be obtained at the highest temperature.
(b) Like CFG and CAC, BO contains a high percentage of carbon. However, it presents more signals associated with different functional groups that could chemically interact with the asphalt binder. This is mainly observed in pyrolyzed BO at the lowest temperature.
(c) The morphological and physicochemical characteristics of BO could benefit the homogeneous distribution in the asphalt binder, making it achieve good storage stability for all the modification percentages evaluated.
(d) Based on the results of the full factorial design, BO can extend the viscoelastic range of the asphalt binder. This means that it tends to increase the rutting resistance, evidenced by higher values of the rutting parameter G*/sin(δ) at high service temperatures, and reduce its Fraas breaking point temperature.
(e) Workability is not greatly affected, since the rotational viscosity values of the asphalt binder increase proportionally to the amount of BO used, implying a slight increase in the mixing and compaction temperature of the hot mix asphalt. When used in the same amount, it also has a similar effect to that caused by CFG and CAC.
(f) The porous structure and chemical characteristics of BO could improve its interaction with the asphalt binder. As a result, resistance to rutting and aging significantly improves proportionally to the amount used. In some cases, it overcomes the effect produced by CFG and CAC.
(g) The application of BO could improve the fatigue resistance of the asphalt binder, but the amount used should be limited depending on the requirements of the Superpave specification for its classification.
(h) BO would help improve the performance of the asphalt binder at low service temperatures through higher strength and deformation capacity than the control asphalt binders. It can be used in all quantities evaluated.

In summary, BO could be used to improve the properties of the asphalt binder at different service temperatures. In this regard, pyrolyzed BO at 300 °C with particle size <20 µm (BO300S) is recommended because it allows for a greater extension of the viscoelastic range of the asphalt binder. In addition, it significantly improves its flow resistance and workability, along with increasing its resistance to rutting and aging depending on the amount applied. In this case, 7.5% BO300S would maximize the benefits at high service temperatures, and good performance would be obtained at low temperatures. On the other hand, using BO could provide a greener solution to improve the strength and durability of asphalt pavements while promoting the circular economy of oats.

## Figures and Tables

**Table 1:** Properties of the asphalt binder used.

Performance Grade	Test Method	Specification	PG 64–22
**Original Binder**			
Flash Point Temperature (°C)	AASHTO T 48 [51]	Min. 230	316
Viscosity at 135 °C (Pa∙s)	AASHTO T 316–19 [52]	Max. 3.0	0.5
Dynamic Shear, G*/sin(δ), 10 rad/s at 64 °C (kPa)	AASHTO T 315 [53]	Min. 1.00	2.22
**Rolling Thin-Film Oven (RTFO)**			
Mass Loss (%)	AASHTO T 240–13 [54]	Max. 1.00	−0.10
Dynamic Shear, G*/sin(δ), 10 rad/s at 64 °C (kPa)	AASHTO T 315 [53]	Min. 2.20	5.59
**Pressure Aging Vessel Residue (PAV**)			
Dynamic Shear, G*∙sin(δ), 10 rad/s at 25 °C (kPa)	AASHTO T 315 [53]	Max. 5000	4833
Creep Stiffness, S at −12 °C (MPa)	AASHTO T 313–12 [55]	Max. 300	265
Creep Stiffness, m-value at −12 °C	AASHTO T 313–12 [55]	Min. 0.300	0.304

**Table 2:** The 2 × 2 × 3 full factorial design for the BO-modified asphalt binder.

Run	Sample	PT (°C)	Particle Size (µm)	Modifier Content (wt% Asphalt Binder)
1	AB-BO300S2.5	300	<20	2.5
2	AB-BO300S5.0	300	<20	5.0
3	AB-BO300S7.5	300	<20	7.5
4	AB-BO300L2.5	300	<75	2.5
5	AB-BO300L5.0	300	<75	5.0
6	AB-BO300L7.5	300	<75	7.5
7	AB-BO500S2.5	500	<20	2.5
8	AB-BO500S5.0	500	<20	5.0
9	AB-BO500S7.5	500	<20	7.5
10	AB-BO500L2.5	500	<75	2.5
11	AB-BO500L5.0	500	<75	5.0
12	AB-BO500L7.5	500	<75	7.5

**Table 3:** Descriptive parameters of particle size distribution.

	BO300S	BO500S	BO300L	BO500L	CFG	CAC
Mean	12.73	4.66	25.29	21.32	23.95	14.20
Median	14.54	5.41	29.15	24.21	25.26	14.91
Modal	17.04	7.07	37.02	41.08	26.46	13.67
Standard deviation	0.39	0.37	0.35	0.41	0.35	0.37

**Table 4:** SEM–EDX microelemental analysis of BO and commercial modifier controls.

Chemical Element	BO300S y BO300L		BO500S y BO500L		CFG		CAC	
	wt%	σ	wt%	σ	wt%	σ	wt%	σ
C	72.10	4.61	78.23	3.70	82.17	15.73	87.09	12.31
O	21.74	2.71	12.85	3.02	8.37	7.01	7.84	4.99
Mg			0.58	0.00	3.41	0.00		
Al					2.50	0.00	0.60	0.00
Si	4.22	2.38	4.27	2.38			9.60	0.00
P			1.83	0.00				
K	1.76	0.32	3.85	2.58			2.50	1.22
Ca	0.56	0.00						
Ti					6.35	0.00		
Cr					3.93	0.00		
Fe					4.61	0.00		
Cu					7.56	0.00		

Note: “σ”: standard deviation.

**Table 5:** SEM–EDX microelemental analysis of AB, AB-0.0, and asphalt binders modified with 5% BO.

Chemical Element	AB		AB-0.0		AB-BO300S5.0		AB-BO300L5.0		AB-BO500S5.0		AB-BO500L5.0	
	wt%	σ	wt%	σ	wt%	σ	wt%	σ	wt%	σ	wt%	σ
C	93.55	0.17	90.83	2.59	89.45	2.92	94.53	0.01	94.23	0.37	94.18	0.28
O	2.72	0.44	3.87	2.35	2.94	1.93	1.60	0.15	1.70	0.47	3.02	0.52
S	4.47	0.54	5.30	0.33	6.56	0.18	3.88	0.16	3.95	0.08	2.81	0.79

Note: “σ”: standard deviation.

**Table 6:** Mixing and compaction temperature as a function of rotational viscosity at 2 poises and 3 poises, respectively.

Sample	Mixing Temperature at 2 Poises (°C)	Compaction Temperature at 3 Poises (°C)
AB	157.7	147.3
AB-0.0	159.4	148.6
AB-BO300S2.5	159.9	148.8
AB-BO300S5.0	160.1	148.9
AB-BO300S7.5	161.1	150.1
AB-CFG5.0	159.2	148.8
AB-CAC5.0	159.8	148.5

## Data Availability

Data are contained within the article.
